# Does the Gut Microbiome of the Insular Lizard *Gallotia galloti* Reflect Variation in Sex, Environment, and Population Genetic Differentiation?

**DOI:** 10.1007/s00248-025-02560-x

**Published:** 2025-06-05

**Authors:** Dayna E. Cottam, Daniel W. Cosgrove, Rodrigo Megía-Palma, Anamarija Žagar, Sara Blázquez-Castro, J. Filipe Faria, Amy E. Turner, Diego O. Silva, Marcio R. Pie

**Affiliations:** 1 Biology Department, Edge Hill University, Ormskirk, Lancashire L39 4QP UK; 2 Department of Biodiversity, Ecology and Evolution, School of Biology, Universidad Complutense de Madrid, Madrid, E-28040 Spain; 3 Department of Ecosystem and Organism Research, National Institute of Biology (NIB), Vecna pot 121, Ljubljana, 1000 Slovenia; 4 Biotechnical Faculty, University of Ljubljana, Jamnikarjeva 101, Ljubljana, 1000 Slovenia; 5 CIBIO-InBIO (Centro de Investigação Em Biodiversidade e Recursos Genéticos), Universidade do Porto, Campus de Vairão, Vairão, 4485-661 Portugal; 6 BIOPOLIS Program in Genomics, Biodiversity and Land Planning, CIBIO, Campus de Vairão, Vairão, 4485-661 Portugal; 7 Departamento de Biologia, Faculdade de Ciências da Universidade do Porto, Porto, 4099-002 Portugal; 8 Genetics & Biodiversity Laboratory (LGBio) - Federal University of Goiás, Goiânia, Goiás Brazil; 9 Universidad de Alcalá (UAH) ES, Pl. de San Diego, s/n, Alcalá de Henares, Madrid 28801 Spain

**Keywords:** Lizard, Island, Canary Islands, Metabarcoding

## Abstract

Despite their critical role in maintaining organismal health, the factors driving intraspecific variation in gut microbiotas in the wild are poorly understood. *Gallotia galloti* is a lizard endemic to the Canary Islands characterized by substantial phenotypic and genetic differentiation across populations, as well as by its ability to occur across considerably different environmental conditions. However, the extent to which such diversity is reflected in their gut microbiota is still unknown. Here, we use metabarcoding of fecal samples to explore how the gut microbiome of *G. galloti* reflects variation in sex, environment, human footprint, and subspecies identity. Fecal samples of 47 individuals were obtained across 13 locations to reflect the extent of intraspecific variation in the species. We found no evidence for consistent differences in microbiota richness across the studied groups, regardless of whether analyses were carried out at the genus, family, or phylum levels. Moreover, neither the richness nor composition of the microbiota was associated with variation in mean annual temperature, annual precipitation, and human footprint. Our results suggest that the generalist diet of *G. galloti* exposes them to a broad range of food items that provide a common template across the island, despite ecological and historical differences between populations.

**Supplementary Information:**

The online version contains supplementary material available at 10.1007/s00248-025-02560-x.

## Introduction

Gut microbiotas play a fundamental role in maintaining organismal health through the facilitation of nutrient uptake, detoxification, and interactions with the immune system [1, 2]. However, studies to date have focused primarily on mammals, and relatively little is known about gut microbiotas of other terrestrial vertebrates [1], particularly squamate reptiles [3–6]. Understanding how gut microbiomes vary across different environments can provide valuable insight into the way they might respond to anthropic pressures, including climate change [7, 8] because the gut microbiota might affect host thermal tolerance [5]. Such studies are particularly important in wild populations, as the composition of the gut microbiota could change in captivity [9, 10].

Current evidence suggests that the drivers of variation in the composition of the gastrointestinal microbiota of wild species might be complex and multifaceted and potentially can act at a variety of temporal and spatial scales. For instance, some studies showed evidence for seasonal variation in gut microbiotas [11, 12], as well as intraspecific variation across habitats [13], which could reflect an opportunistic nature of their composition. On the other hand, some studies have found consistent evidence for historical (phylogenetic) differentiation amongst lineages [14–16], which could indicate that some of those drivers actually take place at several different timescales, with the microbiome composition shifting as ecological and life-history traits diverge over the course of the evolutionary history of each lineage. Likewise, the composition of gut microbiomes might be conserved across populations that are thousands of kilometers (km) apart [17] but also vary amongst depths within single lakes [18]. Identifying the drivers of variation in gut microbiomes is fundamental to understanding how they are assembled, and the corresponding impacts on host organisms in the wild. As a first approximation, factors such as temperature and precipitation could be reasonable potential drivers of variation in the composition of the gut microbiota, not only due to direct effects on the lizard’s physiology and thermoregulation [19] but also indirectly through changes in the vegetation of different sites and the corresponding shifts in available food items [20–23], and yet studies testing them as potential correlates are still limited. Finally, it is also important to note that Tenerife has experienced varied levels of anthropic impacts [24]. If the composition of the gut microbiota of a given species is indeed opportunistic, higher levels of anthropic influence might affect its composition, particularly by homogenizing the taxa across individual hosts based on the bacterial lineages that could be more commonly associated with human surroundings.

**Fig. 1:**
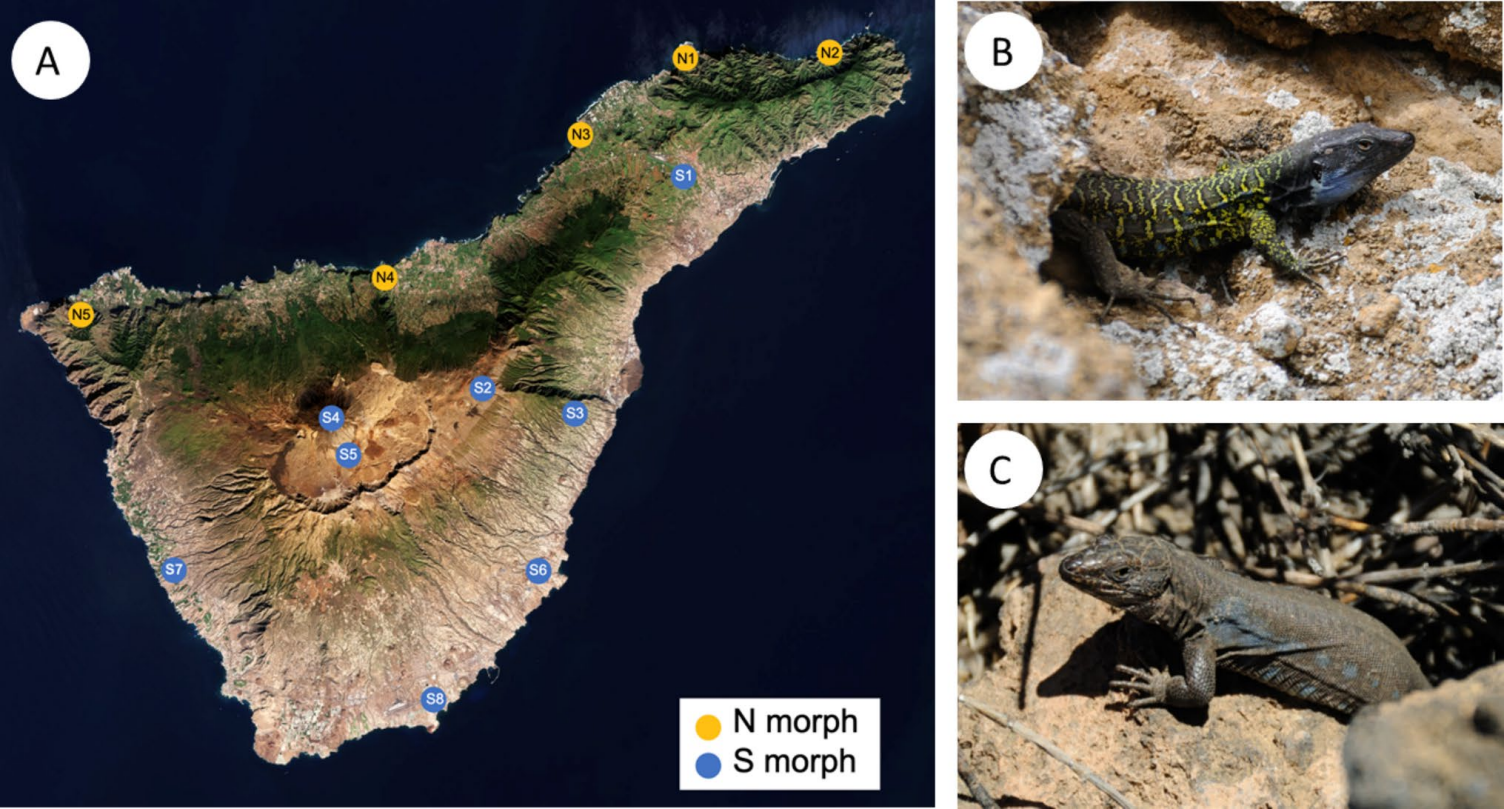
**A** Geographical distribution of the sampled locations in Tenerife, Canary Islands. Samples were obtained across diverse environmental conditions present on the island. Codes indicate either Northern or Southern phenotypes (N morph vs S morph, respectively), according to Brown *et al*. (2016). Symbols correspond to locations as follows: N1: Punta del Hidalgo (*n* = 5); N2: Benijo(*n* = 1); N3: Mirador de Juan Fernández (*n* = 7); N4: La Guancha (*n* = 4); N5: Buenavista del Norte (*n* = 6); S1: La Laguna (*n* = 6); S2: Izaña astronomic observatory (*n* = 2); S3: Puertito de Güímar (*n* = 4); S4: Cono de El Teide (*n* = 1); S5: Base de El Teide (*n* = 4); S6: Porís de Abona (*n* = 3); S7: Barranco de las Moradas (*n* = 2); S8: El Médano (*n* = 2). **B** *Gallotia galloti eisentrauti* (Northern phenotype). **C** *Gallotia galloti galloti* (Southern phenotype). Background image provided by the European Space Agency (CC BY-SA 3.0 IGO)

*Gallotia galloti* is a fascinating species of lizard of the family Lacertidae endemic to the Canary Islands [25]. There are four commonly recognized subspecies [26]: *Gallotia g. eisentrauti* (northern Tenerife), *G. g. galloti* (central and southern Tenerife, including Teide), *G. g. insulanagae* (Roque de Fuera de Anaga, offshore the Macizo de Anaga mountains, northeastern Tenerife), and *G. g. palmae* (La Palma). The populations of *G. galloti* present in Tenerife are found across a remarkable altitudinal range, from sea level to 3715 m of elevation at the top of Mount Teide [27], being distributed across a variety of environments, including coastal shrublands, pine forests, and high-altitude deserts [28]. Such ecological variation is also accompanied by strong phenotypic variation, both in morphology and coloration [29]. Indeed, the phenotypic differences between *G. g. galloti* and *G. g. eisentrauti* seem to reflect both historical (evolutionary) divergence, as well as the result of substantial ecological differences between those regions of the island, with northerly trade winds resulting in the northern slopes (up to a certain altitude) tending to be warm, humid, and lushly vegetated, whereas the south is hot, arid, and barren [30] (Fig. 1). Interestingly, the strong differences in coloration between *G. g. galloti* and *G. g. eisentrauti*, commonly referred to as Northern and Southern morphs, are generally associated with deep genetic divergences, although the congruence between genetic and phenotypic data are not complete [31]. *Gallotia galloti* is also strongly sexually dimorphic, with males being larger and more colorful than females due to their characteristic territorial behavior [32]. Finally, populations across the island are exposed to varying levels of anthropic influence [33], and these disturbances could potentially mask local environmental influences on gut microbiomes. The combination of environmental variation, evolutionary differences between subspecies, and sexual dimorphism may provide a unique opportunity to tease apart the relative contribution of these factors to the composition of the gut microbiome of a squamate lizard.

In this study, we use metabarcoding of fecal samples to explore the extent to which the gut microbiome of *G. galloti* reflects variation in sex, environment, human footprint, and subspecies identity. Given the substantial ecological, phenotypic, and environmental differences present in different populations, we hypothesize that their gut microbiome will have responded, possibly reflecting local evolutionary adaptations. On the other hand, if no evidence for that hypothesis is found, that could indicate that the generalist diet of *G. galloti* exposes them to a broad range of food items that provide a common template across the island, in spite of those ecological and historical differences between populations.

## Materials and Methods

Fresh fecal samples of a total of 47 lizards (29 males and 18 females) were collected across 13 distinct locations in Tenerife during the months of April and May of 2023. The locations were chosen to encompass diverse environmental conditions present in the island, as well as the major phenotypes (Northern and Southern morphs) according to Brown *et al*. (2016) (Fig. 1). Although there is broad agreement between lizard phenotypes and their genetic differentiation, a few populations near the edges of such genetic break sometimes have discrepancies between phenotypes and their mitochondrial lineages, and the causes of such local discrepancies are still poorly understood [31]. However, given that our sampling did not include areas in which there is disagreement between phenotypes and mitochondrial lineages of *G. galloti*, we henceforth refer to them simply as morphs [31]. Samples were obtained noninvasively by applying a slight pressure and massaging the stomach of the lizards, encouraging them to defecate [34]. Each fecal pellet was maintained individually in 1.5-mL Eppendorf tubes filled with ethanol and kept refrigerated until DNA extraction. No animals died or were euthanized during sampling, and all animals were released unharmed at the place of collection. Experimental protocols and research were approved by corresponding authorities of Teide National Park (REGAGE23 s00011246133) and Cabildo Insular of Tenerife (AEI 7/23 - 2023-00252).

DNA extractions were conducted using the ǪIAmp PowerFecal Pro DNA extraction kit (Qiagen, UK), with a few modifications. A FastPrep-24 5G (MP Biomedicals, Santa Ana, CA, USA) homogenizer was used instead of a vortex adaptor to disrupt the mixture of sample and beads; the disruption time was decreased from 10 to 2 min, and the whole process was split into two 1-min cycles at a speed of 9 m/s to prevent DNA degradation. The time spent in the centrifuge between each step was increased from 1 to 2 min to ensure that the pellet structure did not break during movement between centrifuge and pipette transfer. In the final steps, 100 µL of elution solution was used to release the DNA from the filter. Samples were amplified and sequenced by Novogene (UK). Polymerase chain reaction (PCR) was conducted using a Phusion® High-Fidelity PCR Master Mix (New England Biolabs). The 16SV34 region was amplified using a specific forward and reverse primer (F: 5′CCTAYGGGRBGCASCAG′3, R:5′GGACTACNNGGGTATCTAAT′3) [35] and connecting barcodes. The amplified PCR was electrophorized in 2% agarose gels and proper sized products were selected. Equal quantities of the selected PCR products were pooled, subjected to end repair, A-tailing, and subsequently ligated with Illumina adapters. Samples were sequenced on an Illumina platform (Illumina NovaSeq 6000) which generated raw reads of 250 bp.

Paired-end reads were allocated to respective samples using their distinctive barcodes, and then truncated by removing the barcode and primer sequence. The reads were merged using FLASH version 1.2.1.1 [36] (available at http://ccb.jhu.edu/software/FLASH/). High-quality clean tags were obtained by performing quality filtering using the fastp software version 0.23.1 [37]; these tags were then compared to the Silva database (https://www.arb-silva.de/) where chimeras were detected and removed using the vsearch package version 2.16.0 [38] (available at https://github.com/torognes/vsearch). Effective tags were obtained. Denoise was performed on the effective tags using the DADA2 module in ǪIIME2 version ǪIIME2-202202 [39] which obtained Amplicon Sequence Variants (ASVs). The species annotation for each Amplicon Sequence Variant (ASV) was determined by employing ǪIIME2’s classify sklearn algorithm, using a pre-trained Naive Bayes classifier. Unclassified bacteria were removed prior to the analysis. Raw sequence reads were deposited into NCBI’s Short Read Archive under project PRJ A1165502.

Data on mean annual temperature (MAT) and annual precipitation (AP) for each collection site were obtained from WorldClim 2.1 [40]. The human influence (HI) on each population was inferred using the human footprint dataset in QGIS (QGIS.org (2022). QGIS Geographic Information System. Open Source Geospatial Foundation Project. http://qgis.org). It is a global dataset implemented in a geospatial raster and downloaded with 1 km of geographic resolution. It provides HI data normalized by area and biome covering human population pressure (population density), human land use and infrastructure (built-up areas, nighttime lights, land use/land cover), and human access (roads) [41, 42]. Higher HI values indicate higher human development.

Variation in the gut microbiota of *G. galloti* was assessed separately with respect to its richness (i.e., number of distinct ASVs) and composition. The effects of potential correlates on richness, namely MAT, AP, sex, and morph, were assessed using linear mixed models using the lme4 package 1.1–35.5 [43], with sampling site as a random effect. Overall variation in gut bacterial composition between sex and subspecies was investigated using non-metric multidimensional scaling (NMDS) based on Bray-Curtis distances. The effects of discrete (sex, subspecies/morph) and continuous (MAT, AP) predictors on the composition of the gut microbiota were tested using the adonis2 and envfit functions in the vegan package 2.6–4 [44], respectively. All analyses were repeated at the levels of genera, family, and phylum. Unless otherwise stated, all analyses were carried out in R 4.4.1 [45].

## Results

**Fig. 2:**
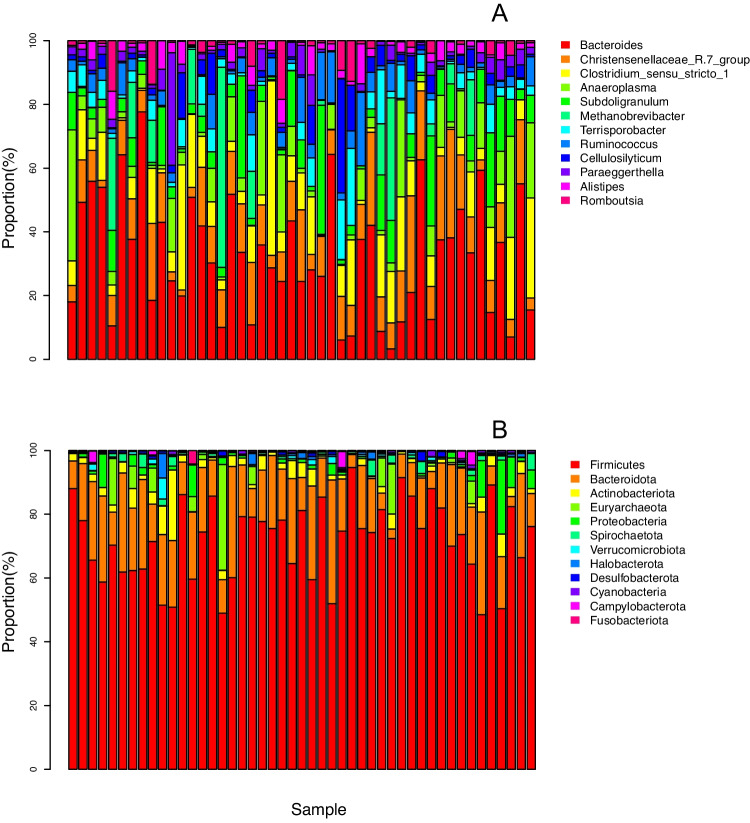
Relative representation of different bacterial taxa (at the phylum (**A**) and family (**B**) level) across the sequenced individuals of *Gallotia galloti*. Samples 1–18 were females, whereas samples 19–47 are males. For visualization purposes, we omitted phyla and families whose representation was below 0.1 and 2% of the total number of reads, respectively

A total of 2,198,765 high-quality reads were obtained across all samples, with an average of 46,782.23 ± 10,510.79 reads per sample (Table S1). After filtering, the final ASV table encompassed 19,164 unique ASVs. The entire dataset included 26 bacterial phyla, although only 5 of them had relative abundance above 1% (Fig. 2A), with Firmicutes and Bacteroidota accounting for over 88% of all reads. At the family level, Lachnospiraceae, Bacteroidaceae, and Ruminococcaceae alone accounted for over half of all reads (Fig. 2B). *Bacteroides* is the most abundant genus, accounting for 21% of the reads across all samples (Fig. S1).

**Fig. 3:**
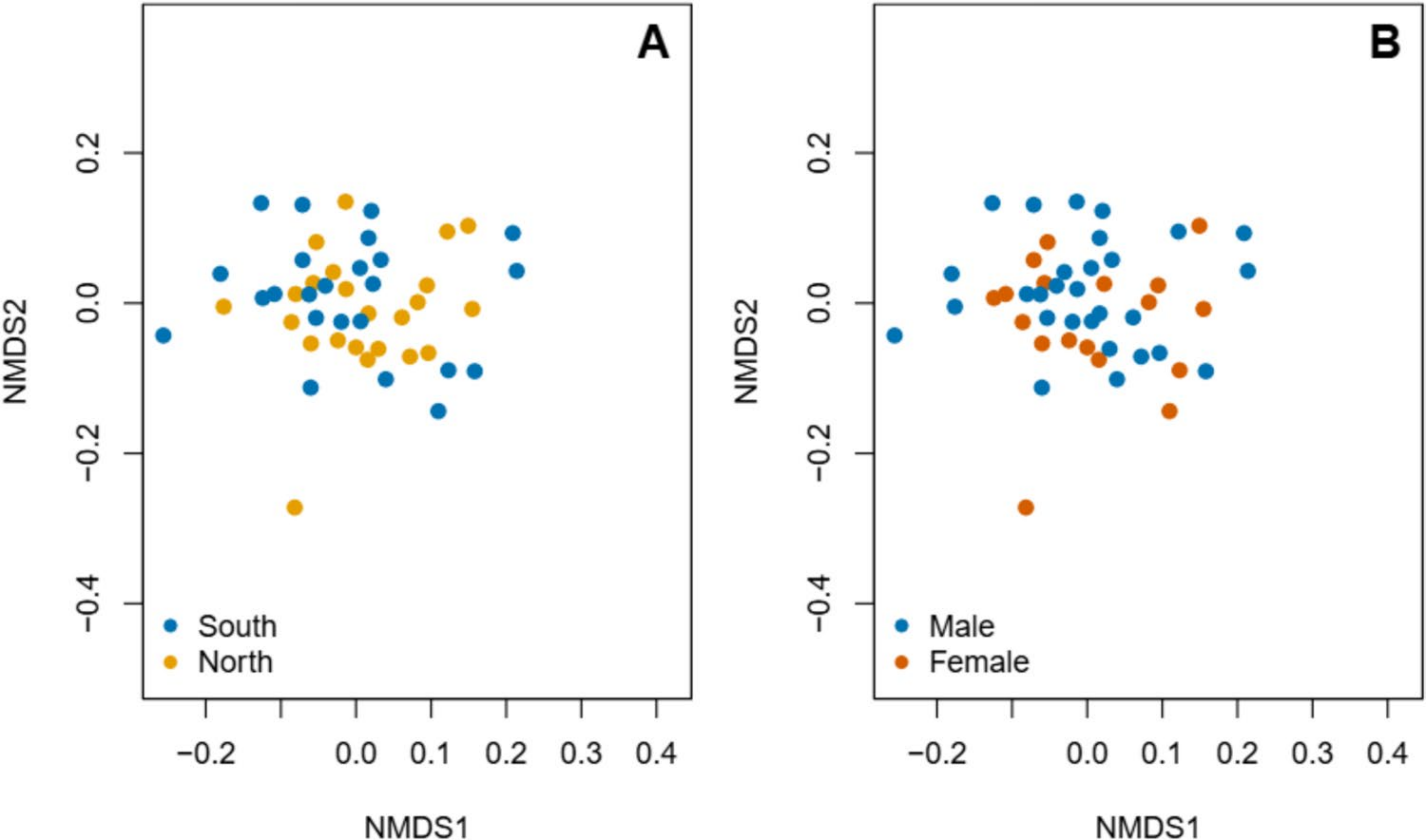
Non-metric multidimensional scaling analyses of the composition of the gut microbiome of *Gallotia galloti*. The underlying data is the same between plots, but they are colored according to sex (**A**) or morph (**B**). See text for details

There were no consistent associations between bacterial composition and ambient temperature, precipitation, sex, morph, or human footprint based on PERMANOVAs, regardless of whether the analysis was carried out at the phylum, family, or genus level (Table 1, Fig. 3). Likewise, when we fit temperature and precipitation data to ordination space using the envfit function, the effects of these environmental variables are also not significant, regardless of taxon (*R*^2^ = 0.02–0.10; *p* = 0.11–0.70). Importantly, there was also no significant effect of any of the variables on the observed richness at any taxonomic level (Table S2).

**Table 1:** PERMANOVA results comparing the composition of the gut microbiota of *Gallotia galloti* between sexes and morphs and ambient temperature and precipitation conditions. Analyses were repeated at the levels of phylum, family, and genus. *MAT* mean annual temperature, *AP* annual precipitation

Taxon	Source of variation	*F*	Df	Df.res	Pr (>*F*)
Phylum richness	(Intercept)	1.2608	1	6.672	0.300
	Sex	0.2274	1	40.156	0.636
	Morph	2.0867	1	9.208	0.182
	AP	0.2087	1	7.238	0.661
	MAT	0.0173	1	6.814	0.899
	HumanFP	2.6782	1	40.774	0.109
Family richness	(Intercept)	39.0486	1	6.133	<0.001***
	Sex	0.1397	1	40.882	0.7106
	Morph	1.3998	1	9.043	0.2670
	AP	0.0302	1	7.019	0.867
	MAT	0.0104	1	6.512	0.922
	HumanFP	0.0081	1	38.921	0.929
Genus richness	(Intercept)	1.3631	1	7.333	0.280
	Sex	0.5379	1	38.793	0.468
	Morph	0.1481	1	9.393	0.709
	AP	0.0439	1	7.637	0.840
	MAT	0.3737	1	7.294	0.560
	HumanFP	0.3737	1	40.675	0.890

## Discussion

In this study, we explored the gut microbiome of the lizard *G. galloti* and tested potential correlates of its composition. The ecology and distribution of an insular species like *G. galloti*, which involves a relatively omnivorous diet with a shift towards herbivory when compared to a typical lacertid diet on the continent [46], could represent relatively similar constraints on their gut microbiome across the island. However, one could reasonably expect that the severe environmental and historical differences between populations could have led to some differentiation.

The general composition of the gut microbiome of *G. galloti* is consistent with previous studies on other lizards [47]. For instance, Firmicutes, Bacteroidetes, and Proteobacteria were the dominant gut microbes of the lizard *Teratoscincus roborowskii* (Sphaerodactylidae), a species from China with a marked herbivore diet, particularly during the summer [12]. Indeed, Firmicutes and Proteobacteria are the bacteria that are most common in lizards when compared to amphibians [47]. *Bacteroides*, the most abundant genus found in *G. galloti*, has been associated with enhanced immune responses in lizards exposed to warming climates by upregulating IFN-β expression in the gut [8]; however, we did not find an association with *Bacteroides* and temperature in our analyses. Its dominance in our samples corroborates the idea that gut microbial communities are likely to comprise a combination of transient, environmentally acquired microbes, commensal “core” microbial taxa, and a flexible pool of beneficial microbes which may confer a selective advantage to the host during periods of stress or rapid adaptation (reviewed in Shapira [48]). Therefore, one of the processes governing the resilience of gut microbiomes to environmental variation could involve the relative importance of these core microbial taxa.

Our results showed no consistent effect of environment (annual temperature and precipitation), subspecies identity/morph, and sex on the corresponding microbiomes. This result is intriguing, given previous studies. For instance, lizard gut microbiota was shown to be strongly affected by their diet, even over relatively short periods of time [4, 49]. Likewise, consistent effects of environmental variables (particularly temperature) have been shown in several studies. For example, a single-generation experimental study in semi-natural conditions demonstrated that warming decreased gut microbiota diversity at 2 months but increased diversity at 13 and 27 months in a desert lizard (*Eremias multiocellata*) [8]. Similar short-term responses were also found experimentally for western fence lizards (*Sceloporus occidentalis* [5]). Finally, Qi *et al*. [50] found substantial differences in the composition of gut bacteria of two species of toad-headed lizards across an altitudinal gradient.

Given the ecological variation across Tenerife in ecological conditions, we would expect that at least some of that variation would affect their diet and, as a consequence, the gut microbiome. On the other hand, Williams *et al*. [7] used population transplantation of *Anolis* lizards from the mainland Panama to a series of warmer islands in the Panama Canal and compared their gut microbiome compositions after three generations of divergence. Although some differences were detected, the microbiome composition was largely resilient to increasing temperatures. These results suggest that, although climate might affect the composition of the gut microbiome, there could be particular situations in which their effect is buffered. Our results suggest that in *G. galloti*, the microbiome is not responding to the diverse climatic conditions of the island, but the nature of this resilience is still poorly understood.

Few studies have explored potential effects of sex on the gut microbiome of lizards. Montoya-Ciriaco *et al*. [51] found no consistent difference between sexes in the gut microbiome of the mesquite lizard *Sceloporus grammicus*. Likewise, in a large comparative study of five *Podarcis* species, host sex had almost no effect on microbiome diversity and structure, with the exception of the proportion of the genus *Corynebacterium*, which significantly differed between sexes in two of the species. Male and female lizards typically show considerable behavioral differences due to variation in foraging and territorial strategies [52]. Our results suggest that the behavioral differences that are commonly observed between males and females of this lizard species [25] are not sufficient to lead to consistent changes in their microbiome composition. This is consistent with previous work that found a lack of sexual trophic niche partitioning in this lizard species [32].

It is important to note that the use of fecal samples does not provide a complete picture of the entire composition of the gastrointestinal microbiota, given that there could be variation between different sections of their gastrointestinal system [4]. However, fecal samples in lizards seem to be largely representative of the hindgut bacterial community [4] and allow for non-destructive sampling of wild populations, overall justifying their use. Another limitation of our study is that we used environmental data from weather stations as opposed to direct measurements of body temperature, which could be affected by the lizard’s thermoregulation strategies. However, weather stations are able to provide more comprehensive characterizations of the climatic conditions in each location, given that they are calculated as averages across the entire year as opposed to direct measurements that could only represent the conditions on the day of collection. Also, we only collected one season and there could be seasonal variation [12]*.* However, given that our samples were obtained over such a short period of time, samples are largely comparable to one another, although sampling in other seasons and neighboring islands in the archipelago might provide interesting insights in future studies. Nevertheless, it is important to note that our sample sizes are likely to not have sufficient power to detect more subtle differences between the tested groups. This is particularly likely if the variation in the gut microbiota that is ecologically relevant to the factors we tested is actually amongst the bacterial lineages that are relatively rare and therefore whose effect might be diluted by the most commonly detected taxa. Future studies, with denser spatial sampling, might be able to uncover these trends if present.

## Conclusion

The original hypothesis of this study predicted that all examined factors would influence the gut microbiome of *G. galloti*. Our results suggest that the generalist diet of *G. galloti* exposes them to a broad range of food items that provide a common template across the island, despite ecological and historical differences between populations. Despite these constraints, this research offers a valuable contribution to our understanding of gut microbiota in wild lizard populations—an area that remains largely underexplored.

## Supplementary Information

Below is the link to the electronic supplementary material.

ESM 1 (DOCX 51.1 KB)

## Data Availability

Sequence data that support the findings of this study have been deposited in GenBank. Please find the Data Availability Statment within the manuscript.
