# Supplementary material for: Does the Gut Microbiome of the Insular Lizard Gallotia galloti Reflect Variation in Sex, Environment, and Population Genetic Differentiation?
Source: Microb Ecol. 2025 Jun 5;88(1):61. doi: 10.1007/s00248-025-02560-x (PMC12141358; doi:10.1007/s00248-025-02560-x)
Supplement: Supplementary file 1 — (DOCX 51.1 KB) [file 248_2025_2560_MOESM1_ESM.docx]

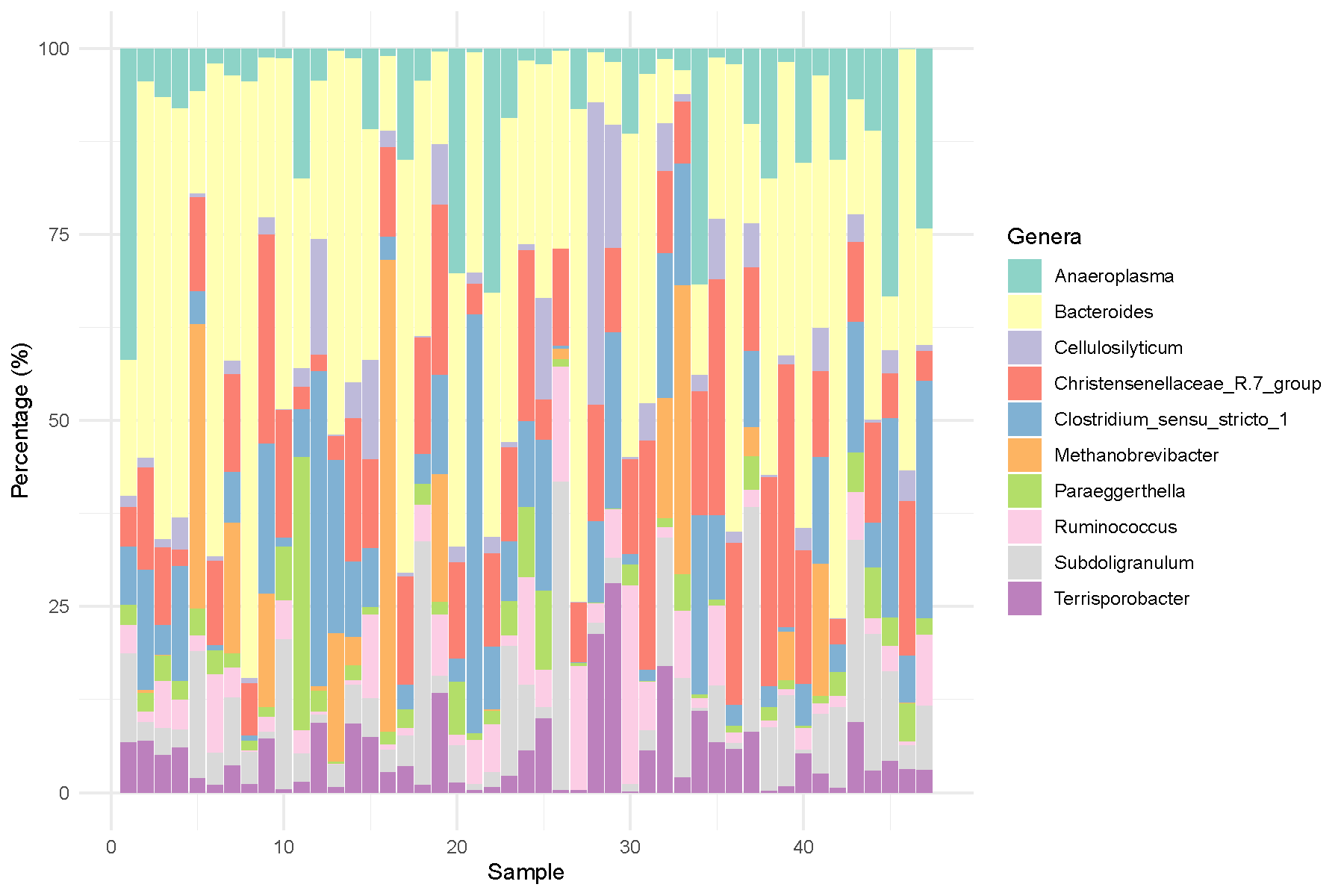


Figure S1. Relative representation of different bacterial genera across the sequenced individuals of *Gallotia Galloti* at the genera level. Samples 1-18 were males, whereas samples 19-47 are females. For visualisation purposes, we omitted genera whose representation was below 2% of the total number of reads, respectively.

Table S1: Summary of sequence processing steps for each sample, including the total input reads, filtered reads, denoised forward and reverse reads, merged reads, and non-chimeric reads. The table presents data for samples S2 to S74, along with the total, average, and standard deviation (SD) across all samples.

| **Sample** | **Input** | **Filtered** | **Denoised Forward** | **Denoised**  **Reverse** | **Merged** | **Non-Chimeric** |
| --- | --- | --- | --- | --- | --- | --- |
| S2 | 64,195 | 59,592 | 57,563 | 57,614 | 52,415 | 30,570 |
| S4 | 66,491 | 61,046 | 59,195 | 59,031 | 53,346 | 32,126 |
| S5 | 72,991 | 68,647 | 67,141 | 67,264 | 62,261 | 59,288 |
| S6 | 64,396 | 60,545 | 59,884 | 60,113 | 58,161 | 56,274 |
| S7 | 65,982 | 62,031 | 61,072 | 60,940 | 57,161 | 54,473 |
| S12 | 63,415 | 58,710 | 56,728 | 57,906 | 51,586 | 27,896 |
| S13 | 65,371 | 60,247 | 57,190 | 58,625 | 50,454 | 32,392 |
| S14 | 66,324 | 61,443 | 60,465 | 59,424 | 55,444 | 32,402 |
| S16 | 64,509 | 60,831 | 59,574 | 59,427 | 53,156 | 49,475 |
| S18 | 65,812 | 61,948 | 60,826 | 60,702 | 55,599 | 51,843 |
| S21 | 66,670 | 62,005 | 60,195 | 59,894 | 54,634 | 33,058 |
| S22 | 71,686 | 66,820 | 65,735 | 65,690 | 62,388 | 58,085 |
| S24 | 63,176 | 58,371 | 55,778 | 56,991 | 48,805 | 32,262 |
| S27 | 70,735 | 67,050 | 66,261 | 66,442 | 63,469 | 61,276 |
| S28 | 59,048 | 55,702 | 54,712 | 54,314 | 49,754 | 46,806 |
| S29 | 62,695 | 59,170 | 58,151 | 57,764 | 54,329 | 51,683 |
| S30 | 63,439 | 58,708 | 57,734 | 58,048 | 54,591 | 35,462 |
| S32 | 65,951 | 62,124 | 60,673 | 60,412 | 53,924 | 48,994 |
| S33 | 65,391 | 61,598 | 60,078 | 59,961 | 54,459 | 50,418 |
| S34 | 64,686 | 60,849 | 60,039 | 59,849 | 56,317 | 52,185 |
| S35 | 65,087 | 61,233 | 60,172 | 60,002 | 55,142 | 49,358 |
| S36 | 62,186 | 58,660 | 57,753 | 57,936 | 55,827 | 54,561 |
| S37 | 62,018 | 58,533 | 57,500 | 57,564 | 53,499 | 50,232 |
| S39 | 63,175 | 59,582 | 58,458 | 58,308 | 53,968 | 49,640 |
| S41 | 69,549 | 65,585 | 64,362 | 64,294 | 59,133 | 56,312 |
| S42 | 65,618 | 61,790 | 60,742 | 60,663 | 56,458 | 53,530 |
| S44 | 66,234 | 62,333 | 61,263 | 61,214 | 55,387 | 52,031 |
| S45 | 63,141 | 58,681 | 56,558 | 55,873 | 50,056 | 27,227 |
| S48 | 66,288 | 62,469 | 61,407 | 61,635 | 57,806 | 53,864 |
| S49 | 64,524 | 61,195 | 60,453 | 60,426 | 57,231 | 51,777 |
| S50 | 64,115 | 59,517 | 58,410 | 57,413 | 53,688 | 36,484 |
| S51 | 66,088 | 62,313 | 61,395 | 61,525 | 57,796 | 55,513 |
| S55 | 66,043 | 61,250 | 59,782 | 58,985 | 53,915 | 48,306 |
| S56 | 63,912 | 59,297 | 57,683 | 57,237 | 52,439 | 33,527 |
| S58 | 65,421 | 61,667 | 60,929 | 61,033 | 58,042 | 54,768 |
| S59 | 70,077 | 65,161 | 62,883 | 63,775 | 55,695 | 35,546 |
| S60 | 63,539 | 59,085 | 58,167 | 58,200 | 54,529 | 51,322 |
| S62 | 47,359 | 44,888 | 43,882 | 43,844 | 39,802 | 36,905 |
| S64 | 61,235 | 57,985 | 57,230 | 57,323 | 55,457 | 54,260 |
| S65 | 62,143 | 58,511 | 57,766 | 57,829 | 55,494 | 53,190 |
| S66 | 72,725 | 68,351 | 67,561 | 67,489 | 64,502 | 60,669 |
| S67 | 64,001 | 59,393 | 58,318 | 57,060 | 52,689 | 34,157 |
| S69 | 75,241 | 71,032 | 69,840 | 70,009 | 65,603 | 61,447 |
| S70 | 71,057 | 66,682 | 65,459 | 65,530 | 60,879 | 58,465 |
| S71 | 66,155 | 61,567 | 60,250 | 60,049 | 56,410 | 35,356 |
| S73 | 66,568 | 61,770 | 60,732 | 59,157 | 54,233 | 35,488 |
| S74 | 65,623 | 61,512 | 60,813 | 60,883 | 58,565 | 57,862 |
| Total | 3,072,085 | 2,877,479 | 2,818,762 | 2,815,667 | 2,606,498 | 2,198,765 |
| Average | 65,363.51 | 61,222.96 | 59,973.66 | 59,907.81 | 55,457.40 | 46,782.23 |
| SD | 4,210.66 | 3,960.62 | 4,002.71 | 4,040.59 | 4,365.45 | 10,510.79 |

Table S2. Generalised linear model of the potential effect of sex, morph, mean annual temperature (MAT) and annual precipitation (AP) on the diversity of the gut microbiota of *Gallotia galloti*. Separate analyses were carried out at the phylum, family, and genus level.

| Taxon | Variable | Estimate | Std. Error | t value | Pr(>\|t\|) |
| --- | --- | --- | --- | --- | --- |
| Phylum | (Intercept) | 0.767841 | 0.835241 | 0.919 | 0.363 |
|  | Sex | -0.10943 | 0.088634 | -1.235 | 0.224 |
|  | Morph | -0.00703 | 0.094253 | -0.075 | 0.941 |
|  | MAT | 0.011331 | 0.025729 | 0.440 | 0.662 |
|  | AP | -0.00011 | 0.001317 | -0.081 | 0.936 |
|  |  |  |  |  |  |
| Family | (Intercept) | 2.119994 | 0.7817 | 2.712 | 0.010 |
|  | Sex | 0.061722 | 0.082952 | 0.744 | 0.461 |
|  | Morph | -0.06498 | 0.088211 | -0.737 | 0.465 |
|  | MAT | 0.006217 | 0.02408 | 0.258 | 0.798 |
|  | AP | 0.000183 | 0.001233 | 0.148 | 0.883 |
|  |  |  |  |  |  |
| Genus | (Intercept) | 2.019328 | 1.007082 | 2.005 | 0.051 |
|  | Sex | 0.111185 | 0.106869 | 1.040 | 0.304 |
|  | Morph | -0.09522 | 0.113644 | -0.838 | 0.407 |
|  | MAT | 0.03008 | 0.031023 | 0.970 | 0.338 |
|  | AP | 0.000903 | 0.001588 | 0.568 | 0.573 |
